# Defocused illumination with beam-shaping condenser for uniform wide-field X-ray imaging

**DOI:** 10.1107/S1600577525011373

**Published:** 2026-01-21

**Authors:** Kuanqiang Zhang, Yijue Luo, Yunfan Di, Chenfei Guo, Changhao Li, Menghui He, Chao Zhang, Fangfang Peng, Wen Liu, Zhao Wu, Yong Guan, Yangchao Tian, Gang Liu

**Affiliations:** a National Synchrotron Radiation Laboratory University of Science and Technology of China Hefei Anhui230029 People’s Republic of China; b Hefei National Laboratory, Hefei, Anhui230031, People’s Republic of China; c Hefei National Research Center for Physical Sciences at the Microscale University of Science and Technology of China Hefei Anhui230026 People’s Republic of China; ESRF – The European Synchrotron, France

**Keywords:** beam-shaping condenser, defocused illumination, diffraction fringes

## Abstract

The beam-shaping condenser (BSC) has become a key optical component in X-ray microscopy, providing a wide field of view with stable and uniform illumination. However, alternating bright and dark fringes often appear at the focal spot, compromising illumination uniformity. To elucidate the formation mechanism of these diffraction fringes, this study conducted theoretical analyses and numerical simulations. Based on this, a defocused illumination approach was developed to alter the focal positions and spatial relative phases of the sub-gratings, thereby enhancing illumination uniformity. The influence of defocused illumination on fringe patterns and intensity distribution was systematically investigated. To validate this approach, a BSC with a Fresnel number of 20 and a 60 µm × 60 µm field of view was fabricated and tested at the soft X-ray imaging beamline of the Hefei Light Source. Experimental results demonstrate that the device achieves uniform top-hat illumination across the full field, effectively suppressing diffraction-induced artifacts. This work offers an efficient and practical approach for achieving wide-field uniform illumination in X-ray microscopy.

## Abstract 2

A defocused illumination method based on a beam-shaping condenser to suppress fringe artifacts arising from rectangular aperture diffraction and sub-grating interference is developed, providing an effective and practical approach for achieving wide-field and uniform illumination in X-ray microscopy.

## 1. Introduction

Full-field transmission X-ray microscopy (TXM) has been widely adopted in materials (Tsai *et al.*, 2022 ▸), biomedicine (Dang *et al.*, 2025 ▸), energy materials (Ding *et al.*, 2024 ▸) and interface dynamics (Chen *et al.*, 2024 ▸) owing to its unique capabilities for non-destructive, three-dimensional imaging at high spatial resolution (Feggeler *et al.*, 2023 ▸). In TXM systems, the performance of focusing optics is critical, as the properties of the focal spot directly determine image quality. Earlier illumination approaches were to use optics such as Fresnel zone plates (Anderson *et al.*, 2000 ▸), ellipsoidal focusing capillaries (Yin *et al.*, 2006 ▸), mirrors (Rau *et al.*, 2005 ▸) or combinations of these devices (Niemann *et al.*, 2003 ▸). However, these optics typically concentrate the beam into a spot of only a few micrometres, smaller than the microscope’s field of view. When imaging intact specimens or large-scale samples, such as biological tissues, this illumination approach cannot cover the full target area and thus fails to capture global information or the complete three-dimensional structure in a single exposure. Consequently, designing and fabricating high-performance, wide-field-of-view focusing optics is a core challenge in TXM development.

To further expand the illumination field, several approaches have been proposed, including beam-shaping condensers (BSCs) (Vogt *et al.*, 2006 ▸), condenser vibration (Tao *et al.*, 2025 ▸), multifocal illumination (Abrahamsson *et al.*, 2017 ▸), lens arrays (Opolka *et al.*, 2021 ▸), structured illumination (Günther *et al.*, 2019 ▸) and field stitching (Fransson *et al.*, 2024 ▸). Among these approaches, the beam-shaping condenser has become an essential component of wide-field X-ray microscopy, as it enables homogeneous and stable illumination over a large field of view and effectively overcomes the limitations of conventional condensers in terms of illumination uniformity and detector matching (Liu *et al.*, 2017 ▸). However, Jefimovs *et al.* found that speckle patterns still appear in the imaging plane of soft X-ray microscopes based on BSC, thereby degrading image quality. This effect arises because the transverse coherence length of the illumination exceeds the size of an individual sub-field, causing diffraction beams from adjacent gratings to interfere at the BSC focal plane (Jefimovs *et al.*, 2008 ▸). To mitigate such illumination inhomogeneity, several strategies have been proposed, including condenser vibration (Vogt *et al.*, 2006 ▸), source size enlargement (Kagoshima *et al.*, 2002 ▸) and sub-field phase optimization (Solak *et al.*, 2002 ▸). Nonetheless, these approaches typically require additional apparatus or complex control, limiting their feasibility for long-term stable operation.

To further enhance illumination uniformity, this study examined the combined influence of rectangular aperture diffraction and sub-grating coherent interference on the illumination profile. Defocused illumination (Gupta *et al.*, 2012 ▸), a commonly employed approach, effectively suppresses inhomogeneities induced by diffraction from rectangular apertures by adjusting the focal positions and relative phase distributions of sub-gratings. Concurrently, coherent interference among sub-gratings produces spatially sensitive high-frequency fringes, the contrast of which can be further mitigated by modulating the optical field distribution. Notably, defocusing generally alters the focal spot size and intensity profile, thereby introducing new constraints. Considering these factors, this study introduces a defocusing-based approach to improve focal spot uniformity, with systematic analysis of its effects on diffraction fringes and intensity distribution, and experimental validation of its performance to evaluate applicability and potential advantages under realistic imaging conditions.

## 2. Simulation and optimization

### 2.1. Formation mechanism of diffraction fringe artifacts

**Figure 1:**
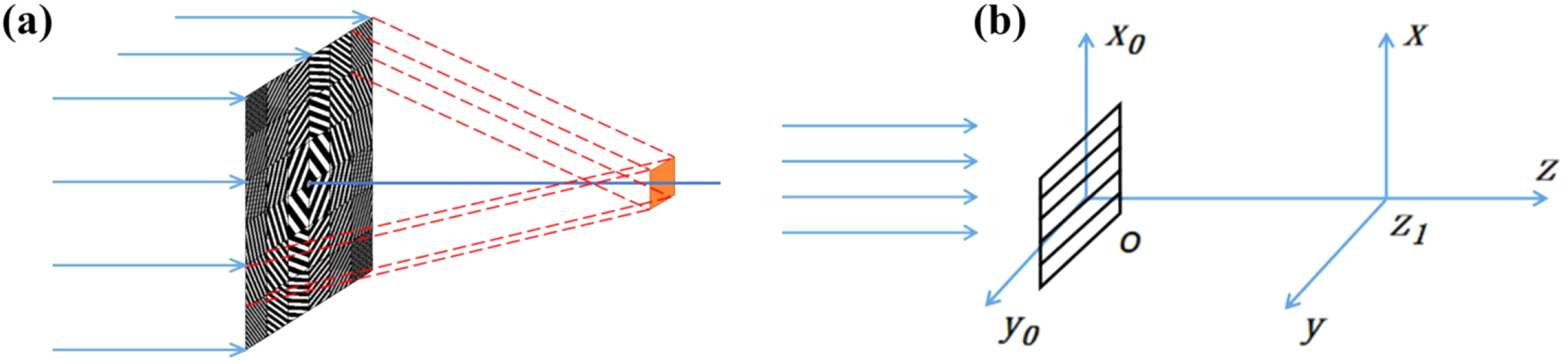
(*a*) Schematic of the beam-shaping condenser focusing principle. (*b*) Illustration of the sub-grating diffraction model.

The beam-shaping condenser is designed based on X-ray diffraction principles, with its focusing mechanism illustrated in Fig. 1 ▸(*a*). By precisely calculating the period and orientation of each sub-grating, the diffracted beams are directed to converge on the same region of the sample, ensuring that the total illumination corresponds to the superposition of all sub-field diffracted beams.

Due to the lateral coherence length of the incident light generally exceeding the spatial scale of individual sub-gratings, the diffracted waves from adjacent sub-gratings coherently interfere, generating diffraction fringes at the focal spot center and reducing its uniformity (Jefimovs *et al.*, 2008 ▸; Vogt *et al.*, 2006 ▸). Furthermore, the basic unit of a BSC consists of a finite-sized array of rectangular apertures, whose intensity distribution exhibits characteristic diffraction patterns and produces pronounced central fringes that affect spot uniformity. The dimensionless Fresnel number (*F*) of a rectangular aperture is defined as *F* = 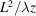, where *L* is the aperture size, λ is the wavelength, and *z* is the distance from the aperture to the observation plane. The Fresnel number directly determines the properties of the diffracted spot. Typically, BSCs are designed with a Fresnel number in the range 1–100 (Jefimovs *et al.*, 2008 ▸; Liu *et al.*, 2017 ▸; Vogt *et al.*, 2006 ▸), inevitably producing pronounced diffraction fringes. Therefore, optimizing illumination uniformity requires a comprehensive consideration of both rectangular-aperture diffraction and subgrating coherent interference.

Under identical BSC structural conditions, the Fresnel numbers for soft and hard X-rays are comparable, both inducing pronounced diffraction fringes. However, the longer wavelength of soft X-rays results in a greater lateral coherence length, leading to more significant diffraction effects. Exploiting this property, numerical simulations and optimization analyses were performed for BSCs in the soft X-ray regime to examine the modulation of diffraction fringes and intensity distribution by defocused illumination. Considering the relatively weaker diffraction effects of hard X-rays, these optimization strategies can be directly extended, thereby enhancing imaging performance across a broader energy range.

### 2.2. Defocused illumination optimizes diffraction fringes

To further investigate the formation mechanism of diffraction fringes, the diffraction patterns of the sub-gratings are calculated using the separated-element method. The diffraction model is illustrated in Fig. 1 ▸(*b*), where the *x*_0_*y*_0_*O* plane represents the diffraction plane and the *xyz*_1_ plane represents the observation plane. Assuming parallel light incidence on the beam-shaping condenser, the complex amplitude 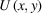 at the observation plane is given by the Fresnel diffraction formula,
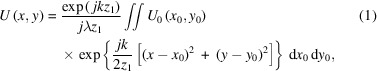
where *U*_0_(*x*_0_, *y*_0_) is the complex amplitude on the diffraction plane, *z*_1_ is the distance between the two planes, λ is the wavelength, and *k* is the wavevector.

**Figure 2:**
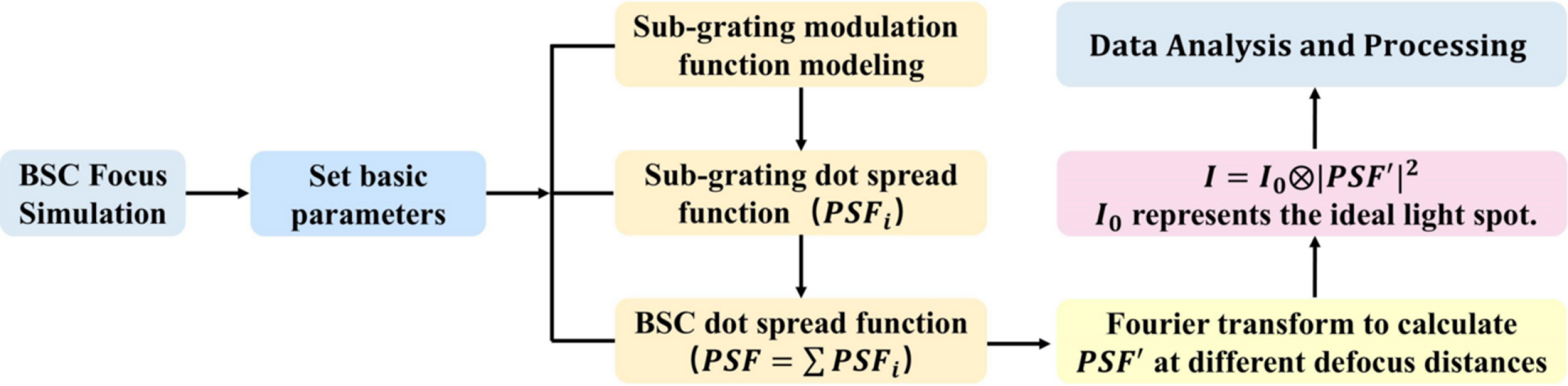
Numerical simulation workflow of the BSC.

**Figure 3:**
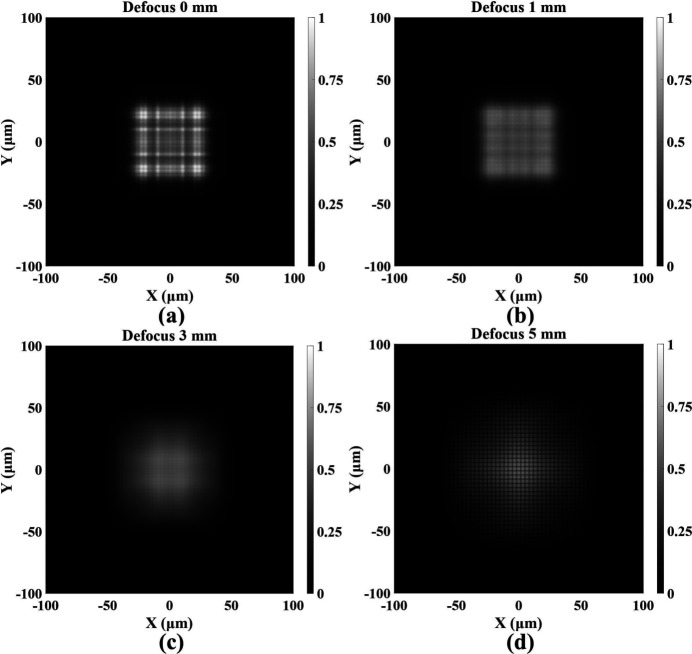
Simulated focal spots obtained at defocus distances of 0, 1, 3 and 5 mm for a secondary light source size of 10 µm × 10 µm.

The condenser consists of multiple square sub-gratings, so the complex amplitude *U*_0_(*x*_0_, *y*_0_) can be expressed as the superposition of contributions from each sub-grating,
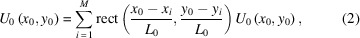
where 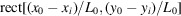 denotes a rectangular function, (*x*_*i*_, *y*_*i*_) is the center coordinate of the *i*th sub-grating, and *L*_0_ is its side length. Accordingly, the complex amplitude at the observation plane expands to
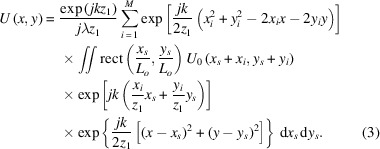
For parallel incidence, the complex amplitude after each sub-grating, *U*_0_(*x*_*s*_ + *x*_*i*_, *y*_*s*_ + *y*_*i*_), equals the modulation function *t*(*x*_*s*_ + *x*_*i*_, *y*_*s*_ + *y*_*i*_). According to the condenser design, each sub-grating is a 1:1 duty-cycle black-and-white grating arranged in a specific pattern. Therefore, the modulation function of each sub-grating can be expressed as
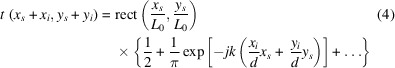
where (*x*_*s*_, *y*_*s*_) denotes the coordinates of an arbitrary point on the (*i*, *j*)th sub-grating in a local coordinate system centered at (*x*_*i*_, *y*_*i*_), and *d* denotes the sub-grating period. The first-order diffracted light from each sub-grating is simulated, and its complex amplitude distribution is
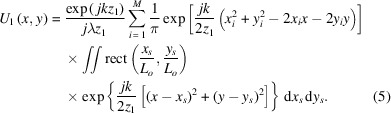
Based on the above equations, a numerical simulation model was constructed in MATLAB, with the simulation workflow shown in Fig. 2 ▸. The key parameters of the beam-shaping condenser are listed in Table 1 ▸, with the Fresnel number of the outermost sub-grating set to 20. Fig. 3 ▸ illustrates the evolution of the focal spot as a function of defocus distance for a secondary light source size of 10 µm × 10 µm.

Simulation results indicate that the diffraction from rectangular apertures and coherent interference among adjacent sub-gratings primarily determine the uniformity of the illumination. As shown in Fig. 3 ▸(*a*), pronounced large-scale fringes can be observed at the center of the focal spot, consistent with the diffraction pattern of a single sub-grating. In this regime, the nonuniformity induced by rectangular-aperture diffraction dominates, resulting in a radial intensity distribution with reduced central brightness. As the detection plane shifts axially away from focus, the focal spots of individual BSC sub-gratings undergo spatial displacement, and the relative phases among sub-gratings are redistributed. This process attenuates the high-contrast fringe artifacts generated by rectangular-aperture diffraction, resulting in a smoother illumination profile and a moderate improvement in field uniformity. Fig. 3 ▸(*b*) demonstrates that introducing a moderate defocus of 1 mm substantially improves illumination uniformity while largely preserving the overall spot size. However, with further defocus, the beam diverges, and the overall planar intensity decreases, ultimately rendering the region unsuitable for flat-top illumination [Figs. 3 ▸(*c*)–3(*d*)].

**Figure 4:**
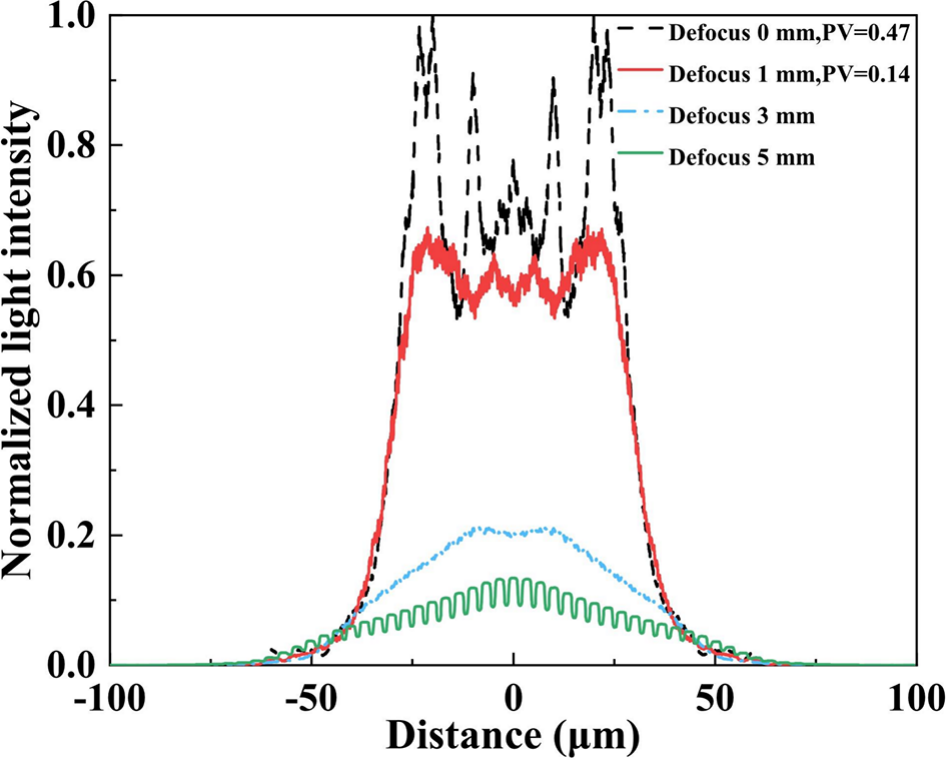
Normalized central line intensity profiles of the simulated focal spot at defocus distances of 0, 1, 3 and 5 mm for a secondary light source size of 10 µm × 10 µm.

To further evaluate the impact of defocused illumination on focal spot uniformity and intensity distribution, normalized intensity profiles along the beam axis were extracted, as shown in Fig. 4 ▸. Compared with the focal plane, a 1 mm defocus markedly improves uniformity, reducing the peak-to-valley values at the spot center from 0.47 to 0.14, while the spot size remains approximately 60 µm × 60 µm and the central intensity shows a slight decrease. However, with further increase in defocus distance, the spot undergoes pronounced divergence, accompanied by rapid intensity attenuation and a transition toward a quasi-Gaussian profile, thereby diminishing its effectiveness for uniform flat-top illumination.

**Figure 5:**
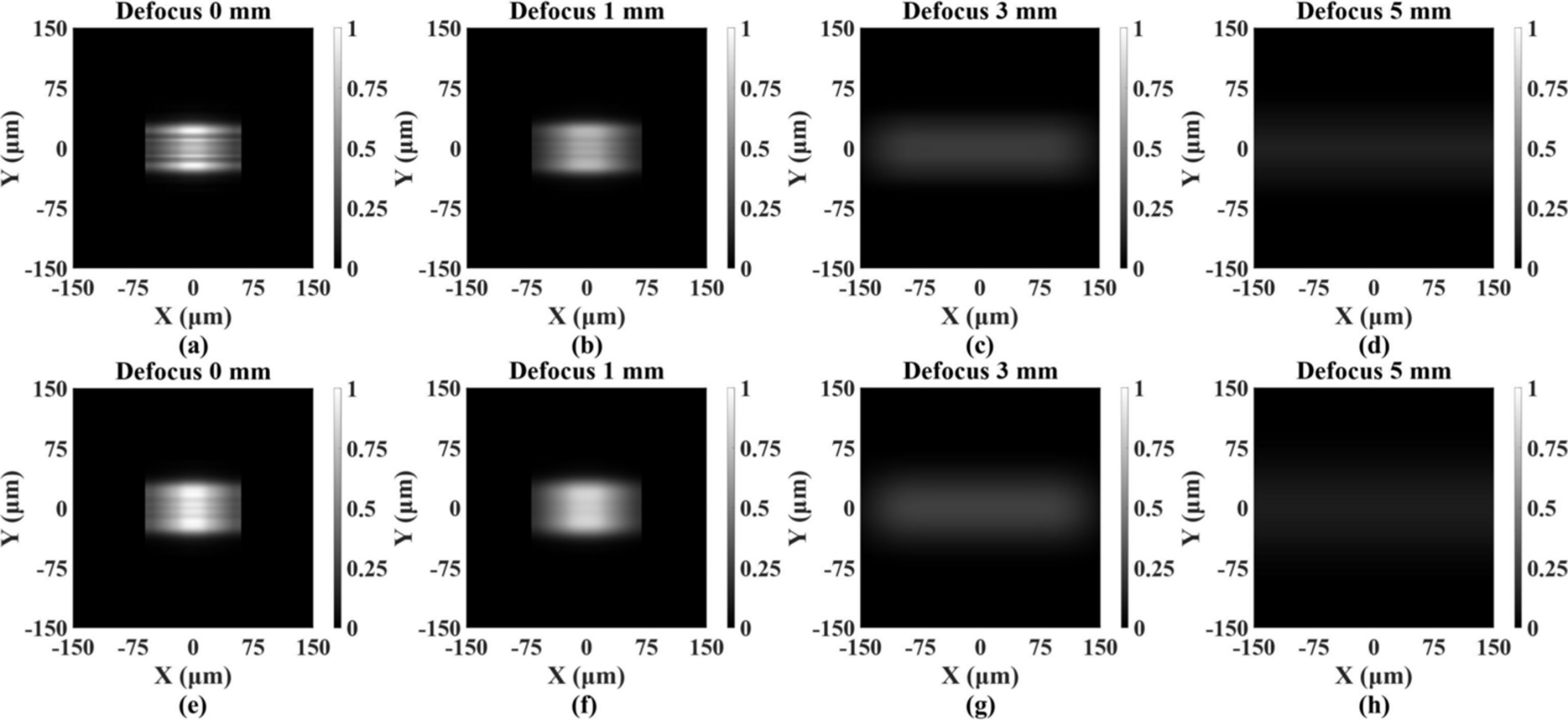
Simulated focal spots at defocus distances of 0, 1, 3 and 5 mm for two secondary light source sizes: (*a*)–(*d*) 400 µm × 20 µm and (*e*)–(*h*) 400 µm × 80 µm.

At the soft X-ray imaging beamline of the Hefei Light Source, the secondary light source was measured to have a lateral size of approximately 400 µm, whereas its longitudinal dimension could be tuned as needed. To assess performance under realistic conditions, numerical simulations were recalculated based on this measured source size, as shown in Fig. 5 ▸.

Simulation results indicate that, owing to the larger horizontal extent of the secondary light source, corresponding to a broader angular distribution, the spot expands significantly in this direction, exceeding the designed 60 µm. At the focal plane, the lateral broadening effectively suppresses diffraction from the rectangular aperture and interference between adjacent gratings, causing the vertical diffraction fringes to vanish almost entirely (Fig. 5 ▸). The focused spot exhibits a longitudinal size of ∼60 µm, accompanied by pronounced large-scale transverse fringes in the central region, which become more pronounced for the smaller secondary light source [Fig. 5 ▸(*a*)]. Introducing a moderate defocus of 1 mm spatially displaces the sub-grating focus and redistributes their relative phase, thereby markedly improving spot uniformity [Figs. 5 ▸(*b*), 5 ▸(*f*)]. However, further increasing the defocus enhances spot divergence and reduces the overall planar intensity, ultimately preventing the maintenance of flat-top illumination [Figs. 5 ▸(*c*)–5(*d*), 5(*g*)–(*h*)].

**Figure 6:**
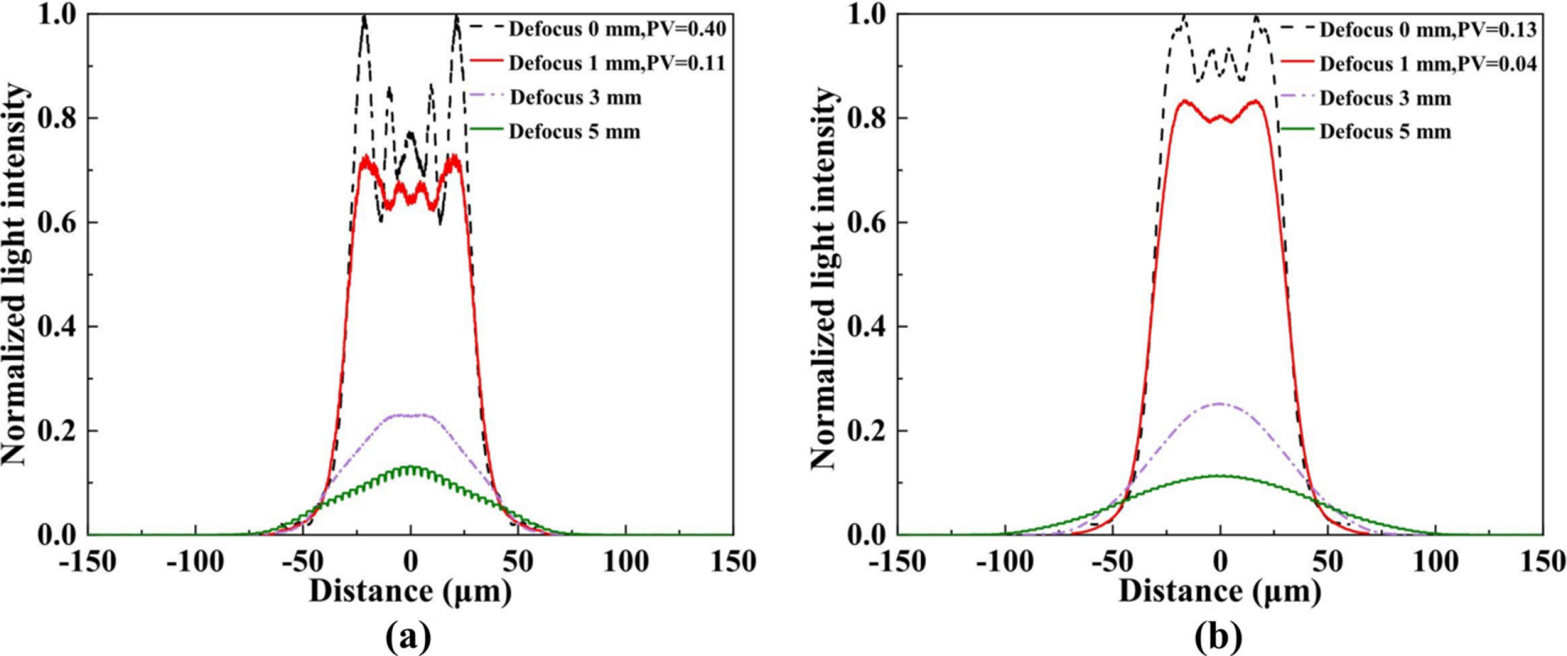
Normalized central line intensity profiles of the simulated focal spot at defocus distances of 0, 1, 3 and 5 mm for two secondary light source sizes: (*a*) 400 µm × 20 µm and (*b*) 400 µm × 80 µm.

To visualize the influence of defocused illumination on spot homogeneity and intensity distribution, the normalized intensity profile along the vertical axis through the spot center was extracted (Fig. 6 ▸). For both secondary-source sizes, a 1 mm defocus produced a pronounced improvement in uniformity compared with the focal plane. For instance, when the secondary source was 400 µm × 20 µm, the peak-to-valley (PV) value in the central region decreased from 0.40 to 0.11. In contrast, a defocus of 3 mm resulted in a substantial increase in lateral spot size and a rapid decay of central intensity, making it difficult to achieve stable, square, uniform illumination. Overall, a 1 mm defocus achieves the optimal balance between spot size and uniformity in this system, providing the most favorable illumination condition.

## 3. Design and fabrication of a wide-field-of-view imaging system

**Figure 7:**
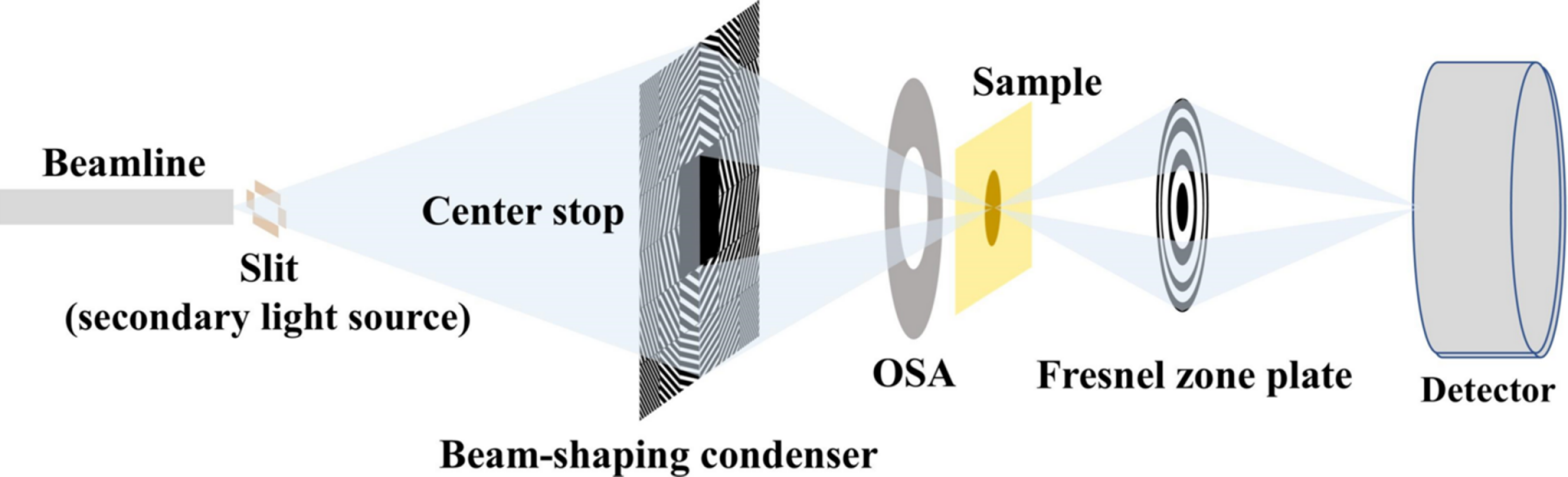
Schematic of the optical path of the wide-field imaging system.

To further validate the suppression of diffraction fringes by defocused illumination using a beam-shaping condenser, we designed and fabricated a wide-field imaging system for the soft X-ray range (Fig. 7 ▸).

Incident X-rays are focused onto the focal plane by a beam-shaping condenser, producing a uniform spot for homogeneous sample illumination. The diffracted light from the sample is then magnified by an imaging zone plate, and the image is recorded by a detector. The beam-shaping condenser has an effective area of 1.8 mm × 1.8 mm, with detailed design parameters listed in Table 1 ▸. To suppress the influence of higher-order diffraction spots and ensure imaging quality, an order-selecting aperture (OSA) with a diameter of 780 µm was incorporated into the setup. The corresponding imaging zone plate has a diameter of 150 µm, an outermost zone width of 100 nm and a focal length of 9.01 mm.

**Figure 8:**
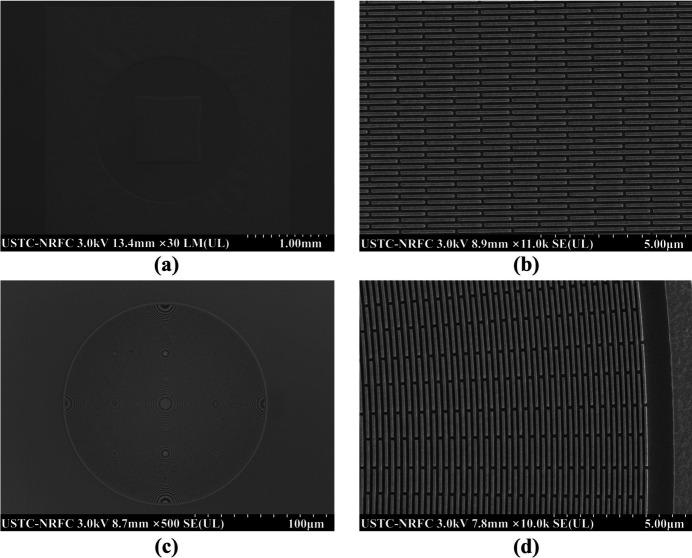
SEM images of the beam-shaping condenser and zone plate. (*a*)–(*b*) Overall and magnified views of the beam shaping condenser. (*c*)–(*d*) Overall and magnified views of the zone plate.

Using a combination of electron-beam lithography and electroplating, we successfully fabricated a gold-based beam-shaping condenser and imaging zone plates according to the design parameters, as shown in Fig. 8 ▸.

## 4. Results and discussion

**Figure 9:**
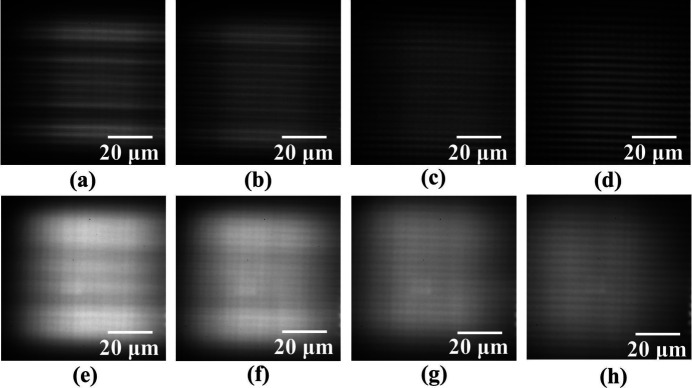
Focal spots at 750 eV recorded on the detector with defocus distances of 0, 1, 3 and 5 mm for two secondary light source sizes: (*a*)–(*d*) 400 µm × 20 µm and (*e*)–(*h*) 400 µm × 80 µm.

The fabricated BSC and imaging zone plate were tested at the BL07W beamline of the Hefei Light Source. To assess the impact of defocused illumination from the beam-shaping condenser on the suppression of diffraction fringes, the BSC was precisely translated along the optical axis to adjust the sample position relative to the focal plane. The results are shown in Fig. 9 ▸.

Due to the large lateral extent of the secondary light source, the horizontal spot exceeded the detector’s effective measurement range, preventing direct acquisition of its complete profile [Figs. 9 ▸(*a*)–9(*h*)]. At the focal plane, the lateral broadening of the secondary light source effectively suppresses diffraction from the rectangular aperture and interference between adjacent gratings, causing the vertical diffraction fringes to vanish almost entirely. The focused spot exhibits a longitudinal size of ∼60 µm, accompanied by pronounced large-scale transverse fringes in the central region, which become more pronounced for the smaller secondary source [Fig. 9 ▸(*a*)]. With moderate defocusing, diffraction fringes are effectively suppressed, and the spot profile becomes increasingly uniform [Figs. 9 ▸(*b*), 9 ▸(*f*)].

**Figure 10:**
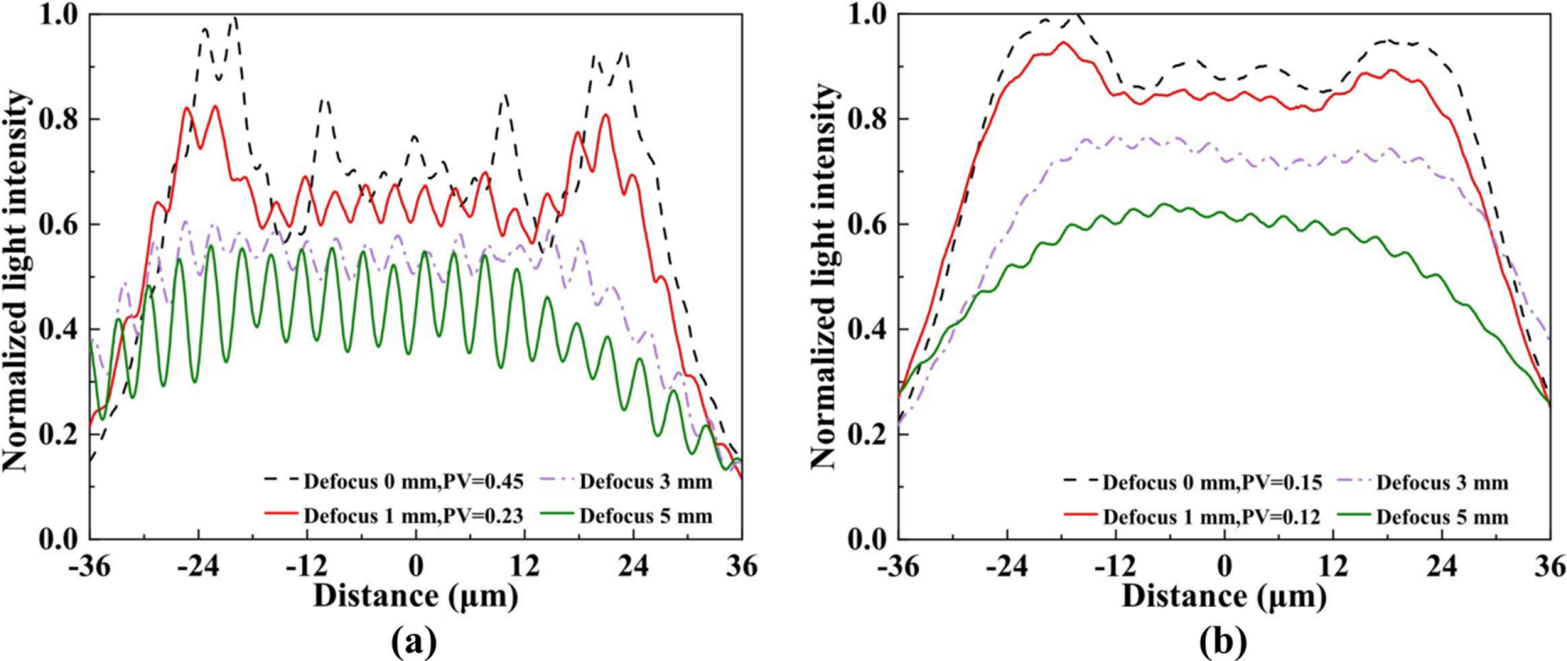
Normalized central line intensity profiles of the focal spots at 750 eV recorded on the detector with defocus distances of 0, 1, 3 and 5 mm for two secondary light source sizes: (*a*) 400 µm × 20 µm and (*b*) 400 µm × 80 µm.

To systematically assess the impact of defocused illumination on the spot field of view and intensity distribution, normalized intensity profiles along the longitudinal cross-section of the spot were extracted [Figs. 10 ▸(*a*)–10(*b*)]. In the experiment, the outer high-numerical-aperture sub-gratings of the BSC were insufficiently illuminated, resulting in missing sub-spots and consequently limiting the beam divergence. As a result, with increasing defocus, the central spot intensity decayed slowly, while the overall uniformity improved. For a secondary source with dimensions of 400 µm × 20 µm, defocusing by 1 mm reduced the PV value in the central region from 0.45 to 0.23, with minor changes in intensity. In contrast, for a source of 400 µm × 80 µm, the PV decreased from 0.15 to 0.12 after the same defocus, while the intensity remained largely unchanged. Further defocusing beyond 3 mm induced pronounced lateral divergence of the spot, resulting in an overall Gaussian-like intensity distribution, which is no longer suitable for flat-top illumination. A comparative analysis of the spots generated by the two secondary sources indicates that the larger secondary light source effectively suppresses rectangular-aperture diffraction and interference between adjacent sub-gratings, rendering defocusing only marginally beneficial for uniformity. Conversely, for the smaller source, moderate defocusing leads to a pronounced enhancement in spot uniformity.

**Figure 11:**
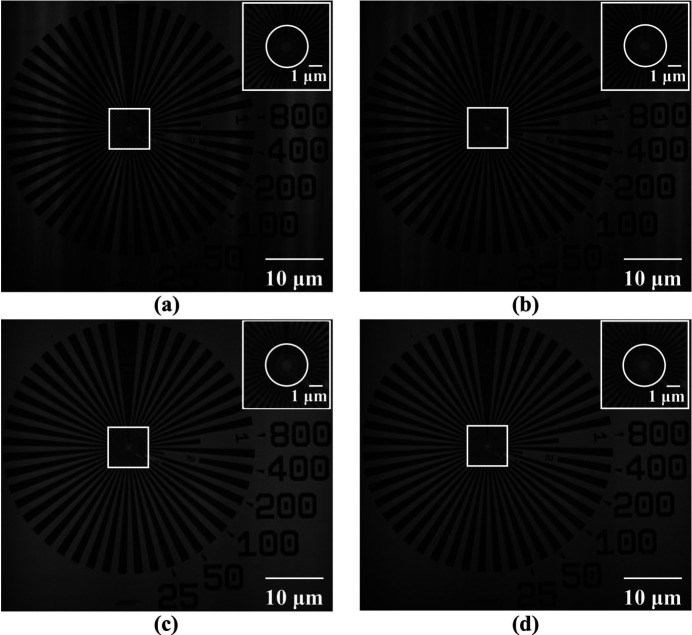
X-ray imaging results with defocus distances of 0 and 1 mm for two secondary light-source sizes: (*a*)–(*b*) 400 µm × 20 µm and (*c*)–(*d*) 400 µm × 80 µm using the beam-shaping condenser.

To further evaluate the imaging performance, a Siemens star was used as the test object. As shown in Fig. 11 ▸, a spatial resolution better than 100 nm was achieved across a 60 µm field of view, delivering high-quality imaging. For the larger secondary source, diffraction fringes exhibited low contrast, with no pronounced patterns observed in the images, and defocusing provided only marginal improvement in spot uniformity. In contrast, for the smaller source, diffraction fringes exhibited pronounced contrast, with clearly visible patterns; moderate defocusing effectively reduced fringe contrast and improved spot uniformity, thereby enhancing imaging quality. These results indicate that BSC-based defocused illumination provides a significant advantage for high-resolution imaging with smaller secondary sources.

## 5. Conclusion

This study systematically investigates the issue of insufficient focal spot uniformity in beam-shaping condensers through a combination of theoretical analysis and numerical simulations. The results reveal that diffraction from rectangular apertures, together with coherent interference between adjacent sub-gratings, generates pronounced diffraction fringes at the focal spot center, an effect particularly significant in the soft X-ray regime. To address this issue, the present study proposes and validates an optimized strategy based on moderate defocusing. Defocused illumination enables the focal spots of sub-gratings to overlap and redistributes their relative phases, thereby substantially enhancing focal spot uniformity. Simulation and experimental results indicate that this approach effectively improves illumination uniformity across a large field of view, providing a viable route toward high-quality three-dimensional imaging. Future research will build upon the developed wide-field imaging platform to perform imaging experiments on large-scale samples such as whole cells and biological tissues, while further optimizing the design of beam-shaping condensers to achieve more efficient uniform illumination and superior imaging performance.

## Figures and Tables

**Table 1:** Simulation parameters

Energy	Field of view	Diameter	Center stop	Outermost zone width
750 eV	60 µm × 60 µm	1.8 mm	720 µm	100 nm

## Data Availability

The data that support the findings of this study are available from the corresponding author on reasonable request.
